# Ethical considerations for engaging frail and seriously ill patients as partners in research: sub-analysis of a systematic review

**DOI:** 10.1186/s40900-021-00254-5

**Published:** 2021-01-31

**Authors:** Claire Ludwig, Ian D. Graham, Josee Lavoie, Wendy Gifford, Dawn Stacey

**Affiliations:** 1 University of Ottawa, Faculty of Health Sciences, School of Nursing, Roger Guindon Hall, 451 Smyth Road, Ottawa, Ontario K1H 8M5 Canada; 2 University of Ottawa, Faculty of Medicine, School of Epidemiology and Public Health, 600 Peter Morand Crescent, Ottawa, K1G 5Z3 Ontario Canada; 3 Ottawa Hospital Research Institute, Clinical Epidemiology Program, 501 Smyth Road, Ottawa, Ontario K1H 8L6 Canada; 4 Geriatric Psychiatry Program, Royal Ottawa Mental Health Centre, 1145 Carling Avenue, Ottawa, Ontario K1Z 7K4 Canada

**Keywords:** Patient engagement, Public and patient involvement, Integrated knowledge translation, Ethics, Research co-production, Patient-partners

## Abstract

**Background:**

The commitment to engage patients as partners in research has been described as a political, moral and ethical imperative. Researchers feel ill-equipped to deal with potential ethical implications of engaging patients as partners. The aim of this study is to identify the ethical considerations related to engaging frail and seriously ill (FSI) patients as partners in research.

**Methods:**

We conducted a sub-analysis of a prior systematic review of 30 studies that engaged FSI patients as partners in research. Studies were included if they reported ethical considerations associated with partnering. We performed deductive content analysis, data were categorized according to Beauchamp and Childress’ Principles of Biomedical Ethics (2019): autonomy, non-maleficence, beneficence, and justice.

**Results:**

Twenty-five studies were included. Common ethical considerations reported in relation to the principles were: *autonomy* – promoting desired level of involvement, addressing relational and intellectual power, facilitating knowledge and understanding of research; *non-maleficence* – protection from financial burden, physical and emotional suffering; *beneficence* – putting things right for others, showing value-added, and supporting patient-partners; and, *justice* – achieving appropriate representation, mutual respect for contributions, and distributing risks and benefits.

**Conclusions:**

When partnering with FSI patients, research teams need to establish shared values and ensure processes are in place to identify and address ethical issues. Researchers and patients should work together to clarify the intent and outcomes of the partnership, actively address power differentials, recognize and minimize the potential for unintended harm, and strive to maximize the benefits of partnership.

**Systematic review registration:**

The protocol for the original systematic review has been registered with the International Prospective Register of Systematic Reviews PROSPERO (CRD42019127994).

## Plain English summary

There are ethical, political and moral reasons to engage patients as partners in research; with evidence to suggest that research produced with patients is more relevant and responsive to their needs. Barriers to engaging patients in research have focused on practical issues but researchers feel poorly prepared to deal with the ethical issues of partnering with patients, particularly those who are frailer and sicker. The aim of this study was to identify ethical considerations related to engaging frail and seriously ill (FSI) patients as partners in research. We conducted a sub-analysis of a recent systematic review of 30 studies that engaged FSI patients as partners in research. Of 30 studies, 25 reported on ethical issues. To enhance autonomy (act independently), common themes were helping patients choose desired level of involvement, addressing power issues, and increasing knowledge and understanding of research. For non-maleficence (cause no harm), common themes were protecting patient-partners from financial burden, physical suffering, and emotional suffering. Beneficence (do good) included putting things right for others, showing value-added, and supporting patient-partners. To enhance justice, three themes were achieving appropriate representation (including across the illness trajectory), mutual respect for contributions, and avoiding over-reliance on individual patients/patient groups or relying on others to speak for FSI patients. When partnering with FSI patients, researchers should ensure processes are in place to identify and address ethical issues. Researchers and patients should work together to clarify intent of the partnership, recognize and minimize the potential for unintended harm, and maximize the benefits of partnership.

## Key points for decision makers

There is mounting pressure on researchers to meaningfully engage patients across the research lifecycle. In spite of increasing evidence on the practical considerations for patient engagement, researchers report feeling ill-equipped to identify and deal with the ethical implications of more intensive and prolonged engagement – particularly with those patients who are frailer and sicker.

This sub-analysis of a prior systematic review identified the ethical considerations of partnering with frail and seriously ill patients in research. Researchers and patients should ensure there are mechanisms in place to identify and pro-actively address ethical issues throughout the course of their partnership. Due consideration must be afforded to mitigating inadvertent harms, maximizing personal benefits to patient-partners, and recognizing and responding to diminished or impaired autonomy so that patients can negotiate their role in the partnership.

## Introduction

Patient engagement in research has been promoted as a political, moral and ethical imperative. It is defined as “the active, meaningful, and collaborative interaction between patients and researchers across all stages of the research process, where research decision making is guided by patients’ contributions as partners, recognizing their specific experiences, values, and expertise” [1] p.682. The practice of patient engagement is driven largely by two overarching justifications [2]. First, it provides a morally acceptable means to an end, with evidence suggesting that it increases the quality and relevance of the research through the incorporation of patients’ unique insights into living with a disease or condition. Second, it is a morally acceptable end in itself, because it is perceived to embed the values and preferences of the community and increase transparency and accountability for the research that is produced [2]. Engaging patients as partners in health research is considered “essential to the ethical foundation of research practice and evidence-based medicine because it ensures that those who have the most important stake in health research will play a significant role in knowledge creation and translation” [3], p.63.

The relevance-based rationale of engaging patients as research partners supports the ethical principle of beneficence, appealing to a focus on the greater good by promoting better patient outcomes [4, 5]. The rationale of promoting transparency and public accountability in research draws on democratic theory and greater citizen involvement, thereby countering medical paternalism and the dominance of ‘expert knowledge’ [4, 6]. Representation and inclusivity function as central tenets of public and patient engagement and are increasingly built into standards to guide practice [7]. Given democratic and relevance-based rationales, patient engagement is now commonly incorporated into the eligibility requirements for receipt of government-funded health research [8, 9].

The growing emphasis on more meaningful and inclusive engagement of patients as partners in research has led to recent reports from researchers who describe feeling ill-equipped to deal with the potential associated ethical concerns [10, 11]. An investigation of early career researchers’ views of patient engagement identified ethical concerns related to: 1) professionalization of patients involved in research (with risks of patients becoming less diverse); 2) adequate remuneration of patients; 3) fair recognition of patients’ experiential knowledge; and 4) tokenism (engaging patients only for symbolic appeal) [10]. In the absence of clear ethical standards to guide the process and to identify and resolve ethical issues as they emerge, current efforts aimed at engaging patients as partners in research run the risk of unintentional harm to those patients involved, particularly those deemed more vulnerable [10–12]. A recent systematic review examining the practical issues and impacts of engaging frail and/or seriously ill (FSI) patients as partners in research established that the vulnerability and frailty of certain patient groups (e.g., frail elderly, patients with high symptom burden from disease/and or treatment, patients with dementia, palliative patients) provides additional complexity to partnering across the research cycle [13]. The practical issues associated with engaging FSI patient-partners may heighten perceived ethical concerns and deter research teams from partnering with them.

### Aim

The aim of this study is to identify the ethical considerations related to engaging frail and seriously ill patients as partners in research.

#### Guiding ethical framework

Given the complexities of competing ethical positions and definitions, Beauchamp and Childress’ principles were utilized as the guiding ethical framework [14]. The framework consists of four broad principles: respect for autonomy, non-maleficence, beneficence, and justice [15]. The principles-based approach suggests a universalizable set of principles for bioethical discourse and is flexible to accommodate local values and practices [16]. It is important to note that primacy is not afforded to any one principle [15].

According to Beauchamp and Childress [15] *respect for autonomy* acknowledges the right for individuals to hold views, make choices, and take actions based on their beliefs and actions. It also means granting individuals the right to privacy and confidentiality. It contains both negative and positive obligations. A positive obligation requires disclosure of information and other factors that allow individuals to make autonomous decisions, (e.g., clinicians and researchers disclose all relevant information to allow the patient-partner to achieve their desired level of involvement). A negative obligation requires that autonomous actions should not be constrained by others and diminish a person’s ability to exercise autonomy (e.g., a patient-partner should not be fearful of alienating a clinician who is providing their treatment(s) and who is an investigator on the study). *Non-maleficence* asserts an obligation to abstain from causing harm to others (e.g., efforts are made to avoid physically or emotionally overburdening patient-partners). *Beneficence* asserts that one must make a positive move to produce some good or benefit for another (e.g., emotional support from the research team may serve as a tangible benefit for patient-partners). *Justice* is based on the notion of fairness and equity. Fairness entails treating all people with equal respect and concern. Equity requires distributing the benefits and burdens of research participation in such a way that no segment of the population is unduly burdened by the harms of research or denied the benefits of the knowledge generated from it (e.g., ensuring appropriate inclusion of diverse patient populations).

The contribution of patients engaged as partners in research (i.e., doing research with) differs significantly from that of a research participant where one is the subject of the study. Patient engagement varies in intensity and takes many different forms e.g., focus groups, surveys, participation in research meetings, membership on advisory boards, and contribution to dissemination activities [17]. Patient engagement also occurs along a continuum, ranging from less intensive engagement (informing or consulting) to more intensive engagement (involving, collaborating, empowering) which is more indicative of partnership [18]. When partnership occurs, there is a shift from researcher as sole expert to one where researchers and patients are both experts, working together to solve problems and co-generate knowledge [19].

## Methods

The systematic review methods were described in detail in the original study and in PROSPERO (CRD42019127994). In summary, a search was conducted of the MEDLINE®, EMBASE®, CINAHL, and PsycINFO databases from journal inception to April 2019. Key words were combined with medical subject headings (MeSH) terms related to “patient engagement”, “patient involvement”, “patient-oriented research”, “integrated knowledge translation,” etc. Two authors (CL, JL) independently reviewed abstracts and full-text articles, extracted data from included articles and conducted quality appraisal of each study (i.e., qualitative, quantitative, and mixed methods). The authors have an appreciation of the ethical issues of partnering with frail and seriously ill patients from lived experience (CL) and clinical practice (JL).

For this sub-analysis, eligible studies from the original review (*N* = 30 studies) had to report on ethical considerations associated with engaging FSI patient-partners in research. Two authors (CL, JL) identified this subset of studies from the original review. Discrepancies were resolved through discussion. Any data relevant to the biomedical ethical framework was extracted from the methods and results sections of included studies.

Two authors independently analyzed all data using deductive content analysis [20, 21]. Content analysis was used to identify themes by: reviewing the texts from included studies, creating themes from the text, and establishing consensus [20]. Themes were then organized under the four principles in the biomedical ethical framework of Beauchamp and Childress: respect for autonomy, non-maleficence, beneficence, and justice [15]. Results were audited by a third author (DS), who has considerable experience of research partnerships with frail and seriously ill patients; discrepancies were resolved through discussion.

## Results

Of the 30 studies in the original systematic review, 25 reported on ethical issues of FSI patient-partners [22–46] (see Table 1 for exemplars).

**Table 1:** Ethical Principles and Themes

Ethical principles & themes	Evidence of ethical considerations reported in studies (exemplars from text)	Frequency (*N* = 25 studies)
**Autonomy**		24 (96%)
Promoting desired level of involvement	- shaping what each person was able and willing to contribute … according to his or her needs and wishes [38]	11 (44%)
	- opportunity to make an informed, non-pressurized decision about whether they would like to be part [27]	
Addressing relational and intellectual power	- group with the co-researchers was facilitated by a staff member, external to the project, as we were concerned that the co-researchers would otherwise avoid potential critical comments [26]	9 (36%)
	- patients (patient-partners) … indicated they felt disadvantaged by their lack of understanding of the complex medical issue [44]	
Facilitating knowledge and understanding of research	- (Patient-partners) received training and support to co-facilitate the focus groups … initial training provided a background to general research methods, specific training on focus group approaches and a discussion on the focus group question schedule [28]	9 (36%)
	- Preparation sessions were held with the co-researchers to orientate them to the study, engage their views about the interview content and structure, and enable them to practice interviewing skills [36]	
Ensuring intentional engagement	- Researchers need to be reflective and transparent about the desired outcomes of their projects and the role of the co-researchers in reaching those outcomes, but also acknowledge … the possibility that initial plans may change [26]	7 (28%)
	- Ensuring that users with dementia are not misled about the nature of their role in the research process [27]	
Guarding against disclosing health and other personal information	- Disclosure of own health cognitive status in an effort to connect with interviewees – publicly affirming their dementia [37]	7 (28%)
	- People’s financial situation is a potentially personal and sensitive issue [26]	
Recognizing and responding to diminishing and impaired autonomy	- cognitive and communication difficulties associated with dementia would have precluded some persons with dementia from taking part, especially those with late-stage dementia [40]	6 (24%)
	- For both co-researchers and participants, we adopted a process model of consent, monitoring and reviewing consent within the context of the research relationship and across the duration of the project [36]	
**Non-maleficence**		20 (80%)
Protecting from financial burden	- … making it cost-free for people to participate, but also considering people’s individual situation [26]	11 (40%)
	- Assistance with travel arrangements was offered, including for an accompanying person for older adults [40]	
Protecting from physical suffering	- Through a shared code between the co-researcher and the experienced researcher (such as the raising of a hand), the co-researcher could indicate when they were fatigued or felt unable to continue with moderating the discussion [29]	11 (40%)
	- meetings should be carefully planned with reference to degree of physical frailty and/or anticipated fluctuations in physical status of co-researchers [39]	
Protecting from emotional suffering	- For some, this direct contact with professionals proved a challenge, perhaps in terms of hearing negative information about one’s own cancer type and potential prognosis, or in the understandable personal emphasis placed on what is discussed in meetings with professionals [23]	7 (28%)
	- emotional cost could be ongoing in terms of revisiting personal experiences through the studies engaged with and then more acutely with a reoccurrence. When this happened individuals often had to withdraw …, as happened for two participants [25]	
Guarding against causing offence	- (patient-partners) were sometimes referred to as professional users or the usual suspects. Having responded to the request to become involved, there was some confusion and annoyance at the use of these divisive terms … being undervalued left some service users feeling undermined and used [23]	2 (8%)
**Beneficence**		14 (56%)
Creating conditions for putting things right for others	- “I am more than willing to put effort into a project if it may benefit my daughter and future generations” [43]	7 (28%)
	- to change things for the better, to be part of shaping new, and more appropriate treatment for others going through a similar experience [23]	
Showing value-added	- Need to demonstrate the value-added nature of its (patient partnership) impact on research processes and outcomes [24]	7 (28%)
	- creation of a feedback loop allowed the patient advisors to understand that their work has meaning and their voices mattered [34]	
Providing support	- one (patient) was admitted to hospital towards the end of the data collection period and later died … a bereavement visit (was conducted) after death by clinician involved in the project [39]	6 (24%)
	- Participants could be open about their difficulties as the (patient-partner) interviewer was seen as someone who understood and shared their problems [36]	
Nurturing opportunities for supplementary benefits	- Being involved has been restorative; it’s given people a role, a job, and a community of interest [37]	5 (20%)
	- providing benefits for the person with dementia including opportunity to exercise skills and abilities [27]	
**Justice**		20 (80%)
Seeking diverse representation	- diverse backgrounds in terms of gender, ethnicity, tumour site and their connection to cancer (patients, carers and others with specific interest in cancer research) [30]	16 (64%)
	- better educated or already engaged in other community efforts, which likely influence their experience and biases [34]	
Ensuring mutual respect for contributions	- respect, reciprocity, and mutual benefit, the essence of collaboration is a vested interest and gain for both parties [43]	12 (48%)
	- There was a perception that participants, and their groups, were peripheral to core activities and priorities [23]	
Distributing risks and benefits	- Same volunteers across multiple projects … may overburden some [22]	4 (16%)
	- Overutilization of willing partners in other studies [38]	

### Respect for autonomy

Six themes were identified under the principle of respect for the autonomy of patients as research partners in 24 (96%) studies [22–45] (see Table 1). Researchers were instrumental in *promoting the desired level of involvement* of the patient-partners [22, 25, 27, 29, 30, 32–34, 36–38] by extending them the courtesy of contributing to the research study according to their willingness and ability [38]. Roles and activities were tailored to the availability, time, and interests of patient-partners [22, 25, 30, 33], with opportunities provided for greater responsibility as they gained confidence and expertise [32, 36]. The need for researchers to be flexible and responsive to changes in patient-partners’ condition was a key facilitator to respecting the autonomy of FSI patient-partners [34, 37] and ensured that patient-partners continued to make informed, non-coerced decisions to initiate or remain partnered in the study [27] including the ability to withdraw effortlessly at any given time [29].

Another theme was *addressing relational and intellectual power* [22, 25–27, 33, 34, 37, 43, 44]. Practical strategies to address relational power differentials included identifying potential patient-partners through a neutral party [27] and external facilitation of meetings to ensure that patient-partners were comfortable to share opinions without influence [26, 34, 37, 43]. One study reported financial compensation may serve as a threat to autonomy because of the potential to shift the perspective of patient-partners, asserting subtle pressure to commit more time on the research project [26]. For intellectual power, sensitivity in the use of complex clinical and academic language is required as it may impede understanding [22, 25, 33, 37, 44] and reinforce power differentials as described in a study where patients with cancer were speaking from a subjective and experiential standpoint in a research environment where objectivity was the accepted mode of working [25].

*Facilitating knowledge and understanding of research* through access to training and education about the research role, research methods and approaches was the third most common theme for autonomy [26, 28, 30–36]. In a dementia study, the academic leads reported a desire to directly support patient partners in applying their own skills and ideas [36].

Respect for autonomy was reported in relation to *ensuring intentional engagement* with transparency in the purpose and expected outcomes of the partnership [22–27, 29]. Studies reported that patient-partners find it difficult to effectively integrate into project activities when their role and purpose lacks clarity [22, 24, 25, 27]. This sometimes required reinforcing the distinction between patients’ roles as research partners versus participants providing data [27]. Written information provided upfront was described as allowing patients to enter into the research partnership in an intentional manner [22].

*Guarding against disclosure of health and other personal information* was another key theme for respecting autonomy [26–29, 36, 37, 45]. Some studies in which patient-partners had a role in conducting research involving direct contact with study participants reported patient-partners had a tendency to disclose personal information to participants diagnosed with cancer [28, 29, 45], impaired cognition, or dementia [36, 37]. Non-verbal disclosure of markedly fragile health status was described in a study where a patient with advanced cancer served as a peer interviewer [45]. Disclosure of personal and potentially sensitive financial information in a group setting striving for consensus on payment of patient-partners was further described as potentially problematic [26].

*Recognizing and responding to diminishing and impaired autonomy* due to deterioration in physical health or cognitive decline was described in studies where FSI patients were partnered in palliative care research [39], dementia research [36, 37, 40], frailty research [41], and cancer studies [42]. Efforts to protect those at highest risk of loss of autonomy included measures such as having clinicians involved in the study familiar with the needs of patients receiving palliative care [39] or ensuring academic researchers were comfortable working with patient-partners with dementia [37]. Initial and ongoing formal consent mechanisms were reported in some studies not only for study participants but for FSI patient-partners [36, 41]. Emerging or severe health and cognition issues were reported as a factor in withdrawal or initial exclusion from the research partnership [40, 42].

### Non-maleficence

Of 25 included studies, 20 (80%) reported ethical considerations related to non-maleficence with four themes focused on protecting patient-partners from financial hardship, physical suffering, emotional suffering, and guarding against causing offence [22, 23, 25, 26, 28–30, 32–39, 41, 43–46] (see Table 1). A common theme was *protection from financial burden* by being a research partner [22, 26, 28, 30, 33, 35, 37, 39, 41, 43, 46]. Efforts to protect patient-partners from negative financial impacts were described as a need to ensure the partnership was cost-neutral for patients with all out-of-pocket expenses compensated (e.g., travel and parking) [33, 35, 37, 39, 46]. Honorariums were provided in some studies to compensate for patient-partners’ contribution, including time spent preparing for meetings, reviewing documentation, and training [30, 33, 43]. The need to address patients’ individual circumstances was reported to ensure some patient-partners were not inadvertently disadvantaged more than others, particularly where costs for support were higher [26], e.g. reimbursement costs for caregivers who were required to escort patient-partners due to physical and cognitive impairments [41]. Costs for out-of-town events (e.g., hotel accommodation) were higher for FSI patient-partners when requiring additional time for rest and recovery [28, 43].

Studies reported the need to *protect FSI patient-partners from physical suffering* [22, 25, 28, 29, 32, 34, 37–39, 43, 44]. Researchers described addressing potential physical discomfort by anticipating and planning activities in accordance with patient-partners’ needs for comfort and accessibility in meeting spaces [34, 37–39] and ensuring activities did not place additional burden on those who were fatigued through illness or treatment [39, 43]. One study reported a pre-arranged signal, such as the raising of a hand, for patient-partners to use when they were fatigued or unable to continue in co-facilitating focus groups [29]. Studies stressed the need for researchers to be able to respond to those with rapidly deteriorating health conditions and to ensure that the practical and physical demands did not exceed the capacity of patient-partners [25, 32, 44].

Non-maleficence also required *protecting patient-partners from emotional suffering* [23, 25, 28, 29, 32, 37, 43]. Studies described the potential emotional impact of patient-partners learning about poor prognoses or unsuccessful treatment outcomes related to their own condition and further stressed the responsibility of researchers to be mindful of how information is presented [23]. Formal debriefing mechanisms were identified as a way to respond to emotional distress, particularly when patient partners had progressive and/or palliative illnesses [25, 29, 37, 43]. One study described the need to ensure that patient-partners were safeguarded from overly bureaucratic attitudes and expressions of powerlessness sometimes expressed by clinicians and researchers [23].

The moral *obligation to guard against causing offence* to patient-partners was reported in two studies [23, 37]. Lack of awareness of certain conditions and lack of confidence in dealing with illness was described as contributing to researchers’ fears of not responding appropriately or potentially offending patients on the research team [37]. Guarding against contributing to a perceived lack of worth or feeling used was reported as essential in mitigating offence to patient-partners, particularly when referred to as the “usual suspects” [23].

### Beneficence

Four themes were identified in 14 (56%) studies under the principle of beneficence and a positive obligation to produce some benefit or good for patient-partners [22–28, 34–39, 43] (see Table 1). Patient-partners described wanting to channel their own experiences with illness and/or with the health system into *putting things right* [22, 23, 25, 26, 34, 35, 43], therefore researchers need to create the conditions to facilitate this occurring. In the face of progressive and debilitating illness, patient-partners described the desire to create something innovative, contribute to new treatments or programs [22, 23], improve existing services [35], and influence positive changes for future generations of patients [43].

Another common subtheme was *demonstrating value-added* of including FSI patient-partners [24–27, 34, 35, 37]. Studies reported having mechanisms for patients to be able to see the positive results of their input, know that their voices mattered, and feel that their work had meaningfully contributed [24, 25, 34]. It was important for patients and researchers to maintain a connection to the lived experience of patients so that tangible positive changes were seen in research processes and patient outcomes [26, 37]. Studies further reported that patients’ input added authority to the findings and generated new insights into the findings [27, 35].

*Providing support* to patient-partners is another theme indicating beneficence [23, 25, 27, 28, 34–39]. FSI patient-partners described great benefits from the supportive relationships that evolved within the group [23, 35], with support sometimes extending beyond the life of the study [38, 39] . The power of peer support within one research group was described as offering a transformative function by empowering patient-partners to embrace an identity that was positively associated with living with dementia and use it in their interactions with others [36].

Feeling supported was a theme related to beneficence that extended to study participants who described an emotional connection with patient-partners conducting research [35]. The emotional connection between patient-partners and participants was further described as providing a sense of safety leading to more candid responses from participants during the interview process [36].

The final theme under the principle of beneficence for FSI patient-partners was *nurturing opportunities for patient-partners to be able to obtain supplementary personal benefits.* These additional benefits were described as restorative by providing patient-partners with new roles, increasing confidence and providing opportunities to learn about research and condition-specific treatments and programs [25, 27, 34, 35, 37].

### Justice

There were three themes about justice reported in 20 (80%) studies [22–24, 26–38, 42, 43, 45, 46] (see Table 1). The most common theme was seeking diverse *representation* among the patient-partners (e.g., gender, age, ethnicity) [26–28, 30, 32, 33, 35, 38, 46]. The need for greater diversity was also reported in relation to socioeconomic status [34, 46] and geography [33]. To achieve diverse representation, studies reported the need to broaden the identification of patient partners through community groups rather than rely on current approaches through treatment centres and support groups [29, 33, 46]. Other disease-specific considerations included different tumour types [26, 28, 30], and patient-partners who were able to represent different stages and severity of illness/disease/condition [27, 28, 33, 40, 46]. A number of studies conflated the experience of frail and/or seriously ill patient-partners with those who are more well (e.g., those with chronic conditions, ex-patients/survivors), members of the public, and caregivers [22, 26, 28, 30, 32–35, 37, 38, 42, 43, 45, 46].

Another theme relevant to justice was ensure *mutual respect for contributions* to the research [22–24, 29, 31, 32, 35] that was described as not expecting patient-partners’ contributions to become over-professionalized [26]. Other studies reported the need to actively address issues of equity within the team [34, 36, 38].

The final theme under justice was *distributing risks and benefits* [22, 34, 35, 38]. Researchers described relying on FSI patient-partners, with whom they had existing relationships, across multiple studies [22, 34, 35, 38] and acknowledged that over-reliance had the potential to place undue burden for research partnerships on individual patients or advisory groups. This practice excluded others from opportunities to contribute to the research produced and the benefits of the partnering process.

## Discussion

The overall aim of our review was to identify ethical considerations related to engaging FSI patients as partners in research. The 25 studies included in this sub-analysis of the larger systematic review provided insight into the ethical challenges facing research teams in partnering with potentially more vulnerable patients, particularly as related to respect for autonomy, non-maleficence, beneficence, and justice. Our findings lead to the following points for discussion.

The nature of complex disease, ageing processes, and aggressive treatment regimens have the potential to be a greater threat to autonomy for FSI patient-partners. Intentional and informed actions assume particular significance in terms of quality of life for patients suffering with disease and/or treatment related symptoms, living with a life-limiting illness, or those dealing with failing cognition. Intentional actions require forethought and knowledge about the series of events that will unfold in the execution of the research study [15]. It is imperative that research teams consider the purpose of partnering with patients, roles, anticipated outcomes (beneficial/harmful), and how these may evolve during the course of the study [26].

Understanding is key for autonomous action [15]. Severity of illness and impaired cognition may inhibit an individual’s full understanding and requires serious consideration during a research partnership. Given bioethical frameworks and practices most familiar to health researchers, the paradigm of informed consent has been more formally recognized by some research teams partnering with those with dementia; whereby, standardized processes for informed consent for patient-partners (similar to that utilized for research participants) have been adopted [36, 47, 48]. Protection of those with developing, impaired or diminished autonomy is a moral obligation for researchers [49] but research partnerships require an approach more analogous to negotiation in order to determine a patient’s initial and ongoing ability and agreement to partner in research. For patient-partners with diminished capacity, a process must be in place to ensure the patient appreciates the extent of their role in the research partnership. Decisions about whether a patient-partner possesses adequate understanding to continue to contribute to the study should be shared between the patient-partner and the principal investigator, thereby allowing the patient-partner to maintain their autonomy whilst ensuring the researcher fulfills their obligation to protect the patient’s well-being.

The potential disparity in relational and intellectual power between the researcher and patient is a significant threat to patient-partner autonomy. Researchers should be particularly mindful of the subtle influences of relational power, especially when selecting their own patients to be patient-partners [50]. Relational power differences may be experienced more acutely by FSI patient-partners who are suffering loss of self-esteem related to illness, loss of function, or altered perception of self; all of which may make them less inclined to share negative experiences so as to preserve the caring relationship with their clinician on the research team [43, 51]. Differences in relational power may potentially silence less confident patients and inhibit their capacity to challenge the ‘professional’ voices around the table [52]. Strategies to reduce relational power differentials and promote participation include providing networking opportunities outside of the study and having meetings facilitated by staff external to the core project team [26]. There is also the risk that some patients may expect to receive preferential treatment if they agree to become project partners, given their direct access to their clinician [51].

Partnering with patients in research has brought new challenges to regulations governing confidentiality and control of information. Disclosure of a patient-partners’ personal information, including diagnoses, to broader audiences during all phases of the research process provides additional challenges related to confidentiality [53]. Patient-partners may not be comfortable publicly disclosing personal details such as those related to their health, financial status, or sexual orientation. Discussions regarding compensation should be made at the level of the individual patient-partners, rather than during group discussion where they may feel uncomfortable to explicitly discuss their preference or go against a consensus decision [26]. Communication of life-limiting illness may also be a very sensitive issue and all parties should be aware of the power of non-verbal cues in signifying severity or advanced stage of illness [45].

There is a propensity to speak to positive impacts rather than the negative impacts of partnering with patients in research [17, 54, 55]. In principle, partnering should be something that is beneficial, or of value to others, and is critical to ethical practice involving patient-partners, but researchers must balance this against potential harms to their patient-partners. Partnering with FSI patients brings additional complexity to this moral obligation. Patients have spoken about the emotional demands of research engagement, particularly during times where they are experiencing treatment effects, exacerbation of illness, or symptoms of disease progression [25]. The impact of finding out about the shortcomings of certain treatments or disappointing results from a clinical trial need to be considered, especially when patient-partners are receiving the same treatment [56]. Dismissive comments from members of the research team about inefficient clinical or bureaucratic processes, or expressions about the competency of colleagues can be potentially upsetting for patient-partners [23].

The lack of diversity among patients partnering in research raises justice-related concerns [4]. Equitable inclusion necessitates attention to diversity in socio-economic, gender and ethnic representation [57, 58]. Partnering with patients who are reflective of the diversity of the larger patient population ensures that research teams are not systematically excluding certain subgroups of patients from having the opportunity to influence research [4]. Ensuring patient-partners are reflective of the broader patient population must also extend to patients who are FSI, such as those with dementia or palliative diseases, or those undergoing acute treatment, who have typically been excluded [13, 29, 59, 60]. The intersection between frailty, serious illness and social determinants of health (e.g., race, socioeconomic status, sexual and gender orientation) demands further critical consideration. The question of “who” are patient-partners has important consequences for which perspectives inform research [61] and raises questions about the rationale and ethical foundation of partnering with patients in research .

Adequate representation requires the inclusion of those who possess the embodied knowledge of having an illness oneself [62]. Yet, there is a tendency towards the conflation of FSI patients with other stakeholders (e.g., patients no longer in receipt of active treatment, caregivers, community or advocacy group members) in the reporting of impact and outcomes of partnering with patients [13]. Policies and guidelines for inclusion of patient-partners compound this issue. The term ‘patient’ is considered an all-encompassing and inclusive label to include those with lived experience of a health concern, as well as caregivers, family members, friends and members of the public [19]. We argue that this definition is fundamentally problematic and a threat to the principle of justice. Conflation of distinct and potentially competing perspectives into the singular term ‘patient’ would appear to be an inherent contradiction of the underlying justification for patient partnerships in research.

There is some evidence that the use of patient advisory groups has assisted in circumventing some of the potential barriers to partnering with FSI patients [22, 24, 30, 33]. While this offers researchers ease and convenience, there is also a risk in over-reliance on such groups [61]. Although caregivers and patient advocacy groups are increasingly called upon as a proxy, arguably they do not fully reflect the range of the patients’ experience, particularly towards end-of-life or during periods of acute illness and/or treatment [63]. Patients’ views shift across the trajectory of illness; lived experience is vastly different following curative treatment for a life-threatening illness, or following stabilization of an acute episodic phase of a chronic condition; the earlier phases of dementia look markedly different than the advanced stages; and approaching death versus a focus on cure or living with a chronic disease brings significantly divergent priorities [27, 64–67]. Additionally, caregivers have their own lived experience of illness, one that is informed by the embodiment of illness in those they are caring for, but also one that is shaped by their caregiving role. The fusion of the patient and caregiver voice as a singular unit is especially problematic given the unique roles that caregivers play, particularly as their loved ones become frailer and sicker [68, 69].

The tendency to unify voices for the sake of expediency does a huge disservice to patients, caregivers and the research community alike, particularly when it comes to distributing the benefits and risks of research. By continuing to privilege the voices of those who speak by proxy, we fail in our efforts to develop and adhere to appropriate ethical guidelines for researchers partnering with FSI patients. We not only fail in meeting the threshold to uphold the principle of justice but also in upholding principles of autonomy, non-maleficence and beneficence. In considering who should speak to the patient experience, it is imperative that researchers consider the goals of the research partnership both in the context of the overall study and with regard to specific activities across the research cycle [70].

There is clearly a need to establish robust ethical standards when partnering with patients in research and to better support researchers in identifying and addressing ethical issues. Our findings further highlight ethical considerations specific to partnering with patients who are FSI. Using an established conceptual framework, such as Beauchamp and Childress’ Bioethical Principles, provides a lens through which to frame the partnership and protect the well-being of vulnerable patient-partners. Researchers and patient-partners can work together to identify and mitigate ethical concerns by: co-defining the purpose and benefits of the partnership, establishing trust and understanding, knowing others’ roles, respecting the right to privacy, creating a safe participatory space, addressing power imbalances within the team and decision-making processes, ensuring participation is revisited and re-negotiated with changes in health or interest, and recognizing contributions (including financial recognition) [10, 13]. Finally, as we build the evidence for ethical practice, it is imperative the process of establishing patients as partners is transparent and we are able to rigorously evaluate the partnership using validated tools, e.g., GRIPP2 [55].

## Limitations and strengths

Contrary to the practical challenges of research conducted with FSI patient-partners, the ethical implications of partnering with FSI were rarely identified as such which meant that there was some degree of subjectivity in how ethical considerations fit within each of the principles of respect for autonomy, non-maleficence, beneficence, and justice. We sought to mitigate this challenge by having two authors review, extract, and independently analyze all data. Discrepancies were resolved by discussion and a third author audited the results. All three authors had a high degree of familiarity with the framework. A further strength of this sub-analysis is the comprehensive search strategy of the original systematic review and identification of studies reporting on FSI patients as partners in research. Although we did not formally evaluate the discrepancies between the reviewers in the deductive content analysis, the primary author (CL) appeared more attuned to issues related to respect. Researchers have an obligation to exercise care in the language used to describe patient-partners, terms such as the “usual suspect” are demeaning to patient-partners [23]. Issues of legitimacy appear to be amplified for those occupying dual role of patient-partner and researcher; considered neither ‘real’ patient, nor ‘real’ academic. The notion of multiple roles is underexplored in the literature on research co-production and clearly warrants additional investigation.

Principlism as an approach to bioethics has been criticized as being reductionist because it ignores information that may not fit neatly into the pre-determined categories [71]. However, the framework offers many practical advantages for application by providing researchers with a common language through which to identify and address bioethical issues. Furthermore, the framework is widely incorporated into medical and health sciences curricula and has been adopted extensively by research institutions for governance and regulation of research [15, 72].

## Conclusion

This sub-analysis of a systematic review adds to an emerging literature base on the ethical considerations of partnering with patients in research [10, 51], specifically as it relates to engaging FSI patient-partners. The involvement of FSI patient-partners has the potential to promote autonomy, provide benefits to all, and ensure their perspective is represented on the research team. Researchers and patients should work together to clarify the intent and outcomes of the partnership, actively address power differentials, recognize and minimize the potential for unintended harm, and strive to maximize the benefits of partnership. Failure to address ethical implications may potentially leave FSI patient-partners at risk of inadvertent harm and deny them benefits that often extend beyond the boundaries of the research study, particularly in terms of feeling supported and restoring an identity beyond that of ‘patient’. However, in order to reap those benefits, FSI patients must be extended the opportunity to partner in research. By having others speak for them, or by excluding them, not only are FSI patients denied the benefits of being involved, they are also denied the benefits of contributing to the direction and outcomes of the very research intended to serve them. The previous review [13] demonstrated that partnering with patients in research is feasible with FSI patients but they must be afforded the opportunity to participate and researchers must create a supportive and accommodating environment that addresses both the practical and ethical considerations of the partnership.

## Data Availability

Not applicable.

## Abbreviations

CINAHL: Cumulative Index to Nursing and Allied Health Literature
EMBASE: Excerpta Medica Database
FSI: Frail and/or Seriously Ill
MEDLINE: Medical Literature Analysis and Retrieval System Online
PROSPERO: Prospective Register of Systematic Reviews
PsycINFO: Psychological Information Database
